# Silent Yet Striking: Medullary Carcinoma Behind an Intestinal Obstruction

**DOI:** 10.7759/cureus.86669

**Published:** 2025-06-24

**Authors:** Nasim Salimiaghdam, Ahmed Mustafa, Irene O Pokuaa, Afshin Hamidi, Emily Chen

**Affiliations:** 1 Internal Medicine Residency Program, Capital Health Regional Medical Center, Trenton, USA; 2 Internal Medicine, Capital Health Regional Medical Center, Trenton, USA; 3 Hematology and Medical Oncology, Capital Health Regional Medical Center, Trenton, USA

**Keywords:** bowel obstruction, colorectal cancer, medullary carcinoma, microsatellite instability (msi-h), mismatch repair deficiency

## Abstract

Carcinoma of the ascending colon, especially the medullary type, is a rare and poorly differentiated form of colorectal cancer. This case report details a case of a 75-year-old woman with a background of cardiovascular issues, hypertension, and dementia who presented with complete large bowel obstruction and was found to have poorly differentiated carcinoma of the ascending colon with medullary features. The surgical approach involved an exploratory laparotomy followed by a right hemicolectomy. The pathological analysis confirmed a pT3N0M0, G3 stage II tumor, characterized by a loss of MLH1 and PMS2 protein expression, indicating microsatellite instability (MSI-H). Since there was no nodal involvement, adjuvant chemotherapy wasn’t deemed necessary. The patient was encouraged to keep up with regular follow-ups, which would include monitoring carcinoembryonic antigen (CEA) levels, a complete metabolic panel (CMP), a complete blood count (CBC), and CT imaging every six months and annually. Although CEA is the most established tumor marker in colorectal cancer, it is still part of the follow-up plan, owing to its lack of sensitivity in the medullary subtype. In addition to this, the recommendation was for a surveillance colonoscopy every three years.

This report sheds light on the case’s pathological, clinical, and follow-up elements, emphasizing the need for personalized patient management.

## Introduction

Colorectal cancer (CRC) continues to be one of the top causes of cancer-related deaths around the globe, with more than 1.9 million new cases diagnosed each year [1]. Medullary carcinoma is a rare subtype of colorectal cancer (CRC) that accounts for less than 5% of CRC [2]. This type is considered a distinct histologic subtype and features poorly differentiated tumor cells, prominent nucleoli, and marked lymphocytic infiltration, and may mimic lymphoma or neuroendocrine tumors [3]. Accurate diagnosis relies profoundly on detailed histopathologic and immunohistochemical evaluation. Unlike conventional adenocarcinomas, medullary carcinomas are often right-sided tumors and have a predilection for older women. They characteristically show reduced expression of CDX2 and CK20, with frequent BRAF V600E mutations [4]. Medullary carcinoma is frequently associated with microsatellite instability (MSI-H), which significantly impacts its prognosis and treatment options. MSI-H arises due to defective mismatch repair (MMR) proteins - commonly MLH1, MSH2, MSH6, or PMS2 - resulting in genomic hypermutability [5]. Medullary carcinoma is often found to be microsatellite unstable (MSI-H). As alluded to above, MSI-H represents a prognostic feature for medullary carcinoma and will also dictate approaches to adjuvant therapy. Patients with MSI-H colorectal cancers are frequently resistant to fluoropyrimidine (eg, 5-fluorouracil) therapy but show a very high response rate to immunotherapy, and nonspecifically PD-1 inhibitors (eg, pembrolizumab). MSI testing should be performed in all newly diagnosed patients with CRC, but should especially be performed in patients with medullary histology [6]. This condition, characterized by issues in DNA mismatch repair, is observed in approximately 15-20% of CRC cases and is associated with more favorable outcomes compared to microsatellite-stable (MSS) tumors [7].

Importantly, lifestyle factors, such as poor dietary quality and low physical activity, are well-established contributors to CRC risk, including right-sided tumors. Diets high in processed foods and low in fiber are particularly implicated in colorectal carcinogenesis. These aspects were relevant to our patient and are discussed further in the case presentation [8].

Managing CRC is a complex process that involves considering various factors, including tumor histology, molecular profile, and the individual characteristics of the patient. Medullary carcinoma, as a distinct MSI-H subtype, often shows better survival rates than typical poorly differentiated adenocarcinomas, even when faced with advanced histopathological features [3]. The best treatment approach for these tumors is still under discussion, particularly regarding the use of adjuvant therapy. Although detailed investigations based on medullary histology are still limited, the larger MSI-H CRC literature supports the positive effects of immunotherapy, particularly when in a metastatic setting. For example, immune checkpoint inhibitors such as pembrolizumab have demonstrated durable responses in MSI-H CRC patients [9]. While we also appreciate that the previous references were related to rectal cancer and neoadjuvant therapy and may not entirely apply to this case, we excluded them for accuracy.

This case report centers on a 75-year-old woman diagnosed with medullary carcinoma of the ascending colon, delving into the pathological, clinical, and prognostic elements of this rare subtype of colorectal cancer.

## Case presentation

The patient is a 75-year-old woman with a complex medical background that includes mild dementia, coronary artery disease (CAD), hypertension, a pacemaker (implanted due to complete third-degree atrioventricular block), and a previous episode of deep vein thrombosis (DVT). She is not bedbound, doesn’t smoke or drink alcohol, does not own any pets, and lives close to her daughter.

Her past surgical history includes a cholecystectomy performed 15 years ago, with no prior abdominal surgeries. She has no known family history of colorectal cancer. Her diet before admission was generally low in fiber and high in processed foods, lacking adequate fruit and vegetable intake. She was overdue for her screening colonoscopy and had never undergone colorectal cancer screening.

Before presentation, the patient was independent in basic activities of daily living but had declining nutritional intake over recent months, likely contributing to a worsened physiological reserve. She had also experienced unintentional weight loss, microcytic anemia, and progressive fatigue, which prompted outpatient evaluation. Laboratory results revealed microcytic anemia, raising early concern for gastrointestinal blood loss or malignancy.

She presented to the emergency department with two days of worsening abdominal distension and sharp, colicky pain localized to a bulge in the right lower quadrant that had previously been reducible. The current hernia was large, irreducible, and tender to palpation, raising concern for incarceration and possible bowel compromise. Her vital signs on admission were: BP 102/68 mmHg, HR 92 bpm, Temp 98.4 °F, RR 18, and SpO2 96% on room air. Her Karnofsky Performance Status (KPS) was 50%, and ECOG performance status (PS) was 3, reflecting limited self-care capacity.

Laboratory investigations revealed WBC 13,000 per microliter (μL), hemoglobin 11.2 grams per deciliter (g/dL), platelets 320,000 per microliter (μL), creatinine 1.0 milligrams per deciliter (mg/dL), and lactate 2.1 millimoles per liter (mmol/L).

**Figure 1:**
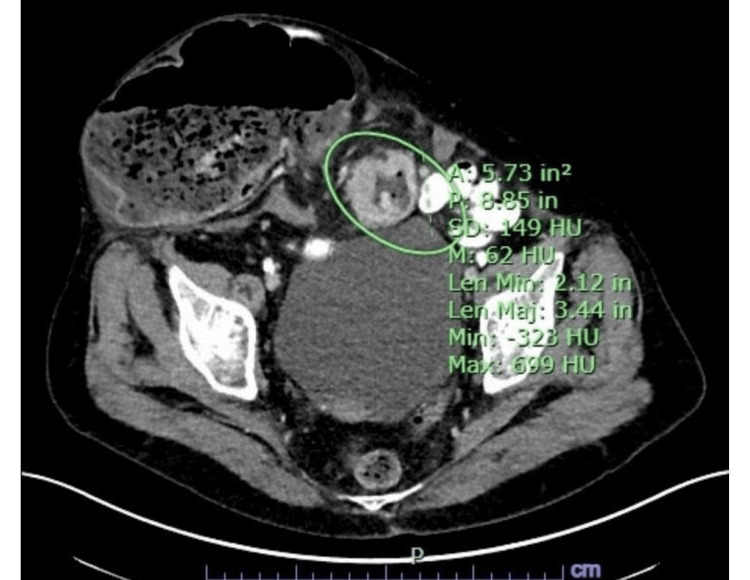
Axial CT scan showing distended cecum and air-fluid levels, indicating significant bowel obstruction

**Figure 2:**
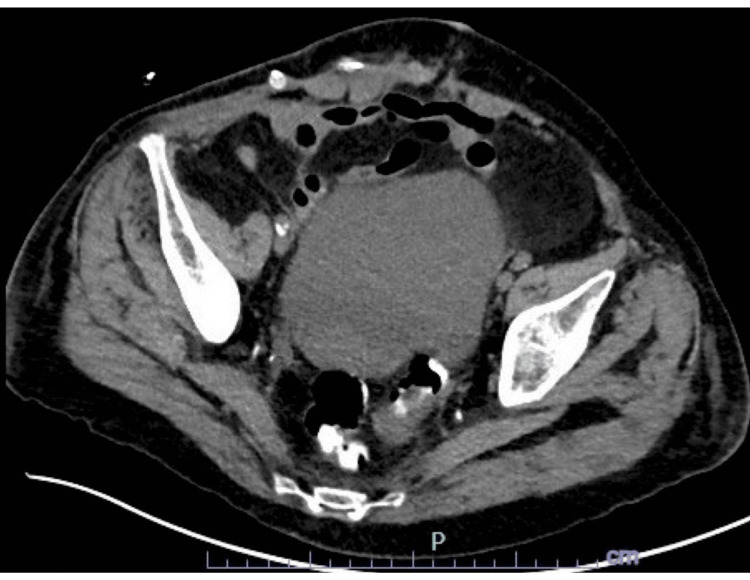
Coronal CT image showing an incarcerated hernia sac contributing to bowel obstruction

After evaluation, CT imaging demonstrated a markedly distended cecum with air-fluid levels, consistent with a large bowel obstruction (Figure 1), as well as a coronal view revealing an incarcerated hernia sac contributing to the obstruction (Figure 2).

An urgent exploratory laparotomy was performed, revealing an incarcerated right inguinal hernia with an ischemic cecum and proximal ascending colon. There was no gross perforation or peritonitis. A frozen section from the affected bowel suggested high-grade dysplasia. Given these findings, the surgical team proceeded with a right hemicolectomy and primary anastomosis with regional lymphadenectomy for oncologic evaluation. A total of 17 lymph nodes were removed. The rationale for such an extensive procedure was based on the intraoperative suspicion of malignancy, confirmed by the frozen section.

Thankfully, her recovery after surgery went smoothly, and she was able to return to a soft, low-residue diet initially, which was advanced gradually as tolerated. A registered dietician was consulted to provide nutritional counseling to the patient and her daughter, emphasizing a high-fiber, plant-forward diet rich in whole grains, vegetables, and omega-3 fatty acids to support gut health and reduce the risk of recurrence. Additionally, the care team discussed light, regular physical activity with the patient and her family to promote gastrointestinal motility and enhance recovery.

A histopathological examination of the removed tissue showed an invasive, poorly differentiated adenocarcinoma with medullary differentiation consisting of solid sheets of malignant cells, excessive tumor-infiltrating lymphocytes, and a syncytial growth pattern. These histological findings are consistent with medullary carcinoma and distinguish it from other high-grade colorectal tumors measuring 6.7 cm (G3). The tumor had invaded the subserosal fat tissue within 1 mm of the serosal surface. There was lymphovascular invasion but no signs of perineural invasion. The surgical margins were clear, and all 17 lymph nodes examined were metastasis-free. Immunohistochemical staining revealed a loss of MLH1 and PMS2 protein expression, indicating microsatellite instability (MSI-H). Based on these pathological findings, the tumor was classified as pT3N0M0 (Stage II).

While medullary carcinoma of the colon does not typically present with unique symptoms, this case reflects the common initial features seen in colorectal malignancy: abdominal pain, anemia, and obstructive symptoms. The diagnosis is often incidental or made during evaluation for complications such as obstruction or hernia [10].

**Figure 3:**
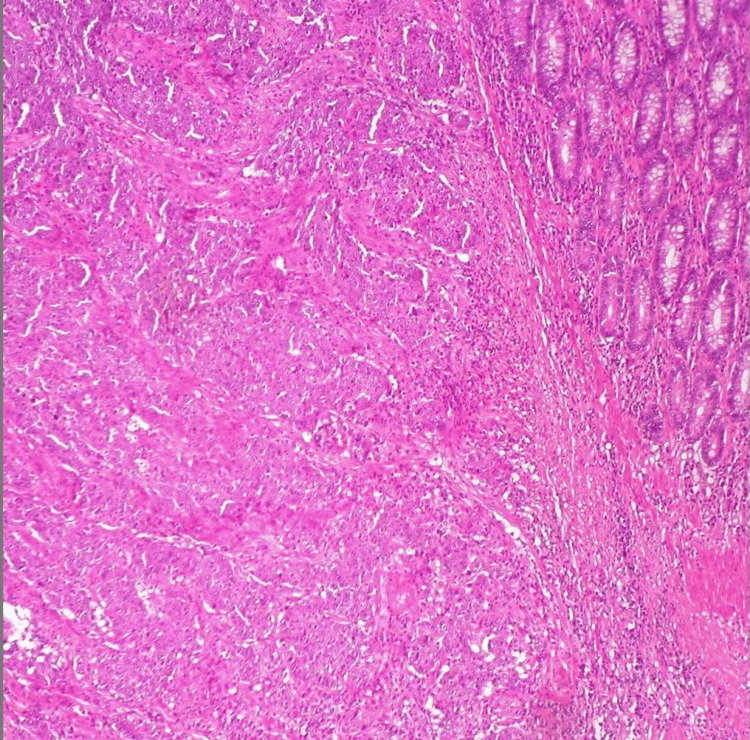
Histopathological section of medullary carcinoma of the colon (H&E stain, ×200 magnification)

**Figure 4:**
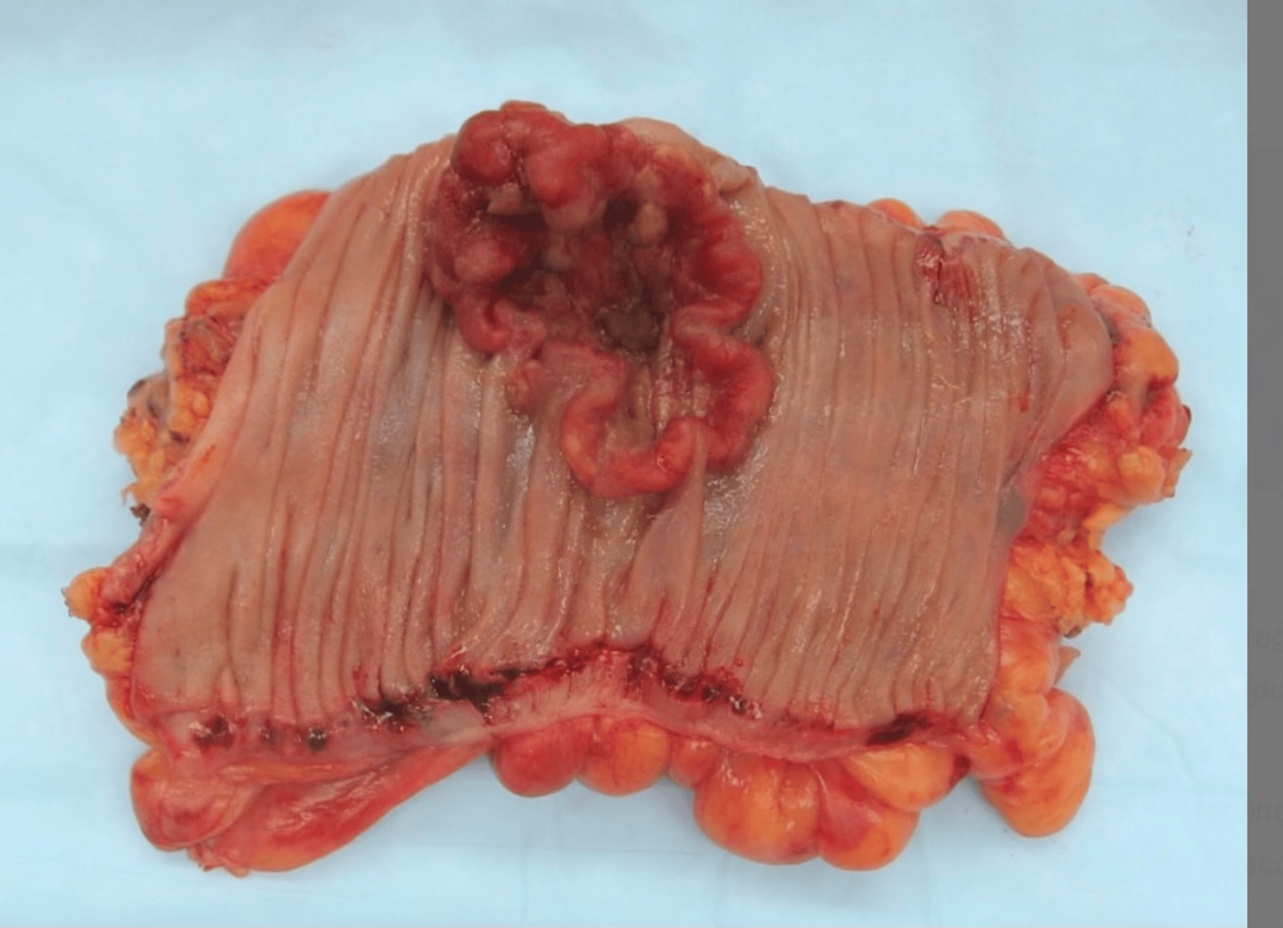
Gross surgical specimen of the resected cecal medullary carcinoma

Gross pathological images and H&E staining demonstrated a diffuse infiltration of pleomorphic malignant cells with prominent nucleoli and minimal glandular formation, consistent with medullary differentiation. Immunohistochemical analysis confirmed the loss of MLH1 and PMS2 with preserved MSH2 and MSH6, supporting the MSI-H phenotype. Intraoperative and pathological images have been included (Figures 3-4).

The patient had an uneventful postoperative recovery and was discharged on postoperative day 7. At the nine-month follow-up, she remained alive, in stable condition, and without evidence of disease recurrence. A registered dietician was consulted to provide nutritional counseling to the patient and her daughter, emphasizing a high-fiber, plant-forward diet rich in whole grains, vegetables, and omega-3 fatty acids to support gut health and reduce the risk of recurrence. Additionally, the care team discussed light, regular physical activity with the patient and her family to promote gastrointestinal motility and enhance recovery.

## Discussion

Medullary carcinoma of the colon is an unusual but increasingly recognized subtype of colorectal cancer that has unique morphological and molecular characteristics. Medullary carcinoma possesses solid architecture with sporadic or very poor gland formation and dense lymphocytic infiltrate. Its morphology may resemble other cancers, such as lymphoma or poorly differentiated neuroendocrine tumors, and for this reason, definitive histological classification is essential. In addition, because of the frequent association of medullary carcinoma with MSI-H, it has a more favorable prognosis compared to canonical colorectal cancers and less susceptibility to fluoropyrimidine-based chemotherapy [11,12].

A key feature of medullary carcinomas is the presence of microsatellite instability-high (MSI-H), which not only helps in diagnosing the condition but also has important implications for treatment. Microsatellites are short, repetitive sequences of DNA scattered throughout the genome that are particularly prone to replication errors in the absence of proper DNA mismatch repair [13]. MSI-H indicates a defective DNA mismatch repair system, which in turn gives rise to genomic hypermutability. This MSI-H phenotype is prognostically and therapeutically relevant: it has been linked to favorable long-term outcomes in CRC patients and better responses to immunotherapy. Therefore, MSI testing is now part of the standard of care for all CRC cases, particularly those with high-grade or atypical histologic features, like medullary carcinoma [14]. Research indicates that patients with MSI-H colorectal cancer often benefit from better survival rates, even when faced with high-grade histopathological features. These tumors are also more likely to respond well to immunotherapies like pembrolizumab, an anti-PD-1 monoclonal antibody that has shown effectiveness in treating metastatic MSI-H colorectal cancer [15,16].

The diagnosis of medullary carcinoma requires histopathological examination, typically from surgical resection or an excisional biopsy specimen. “Observation,” as used here, refers to close postoperative monitoring without immediate adjuvant therapy, particularly in stage II MSI-H tumors where traditional chemotherapy may be ineffective and immunotherapy may be deferred based on risk stratification [17,18].

**Figure 5:**
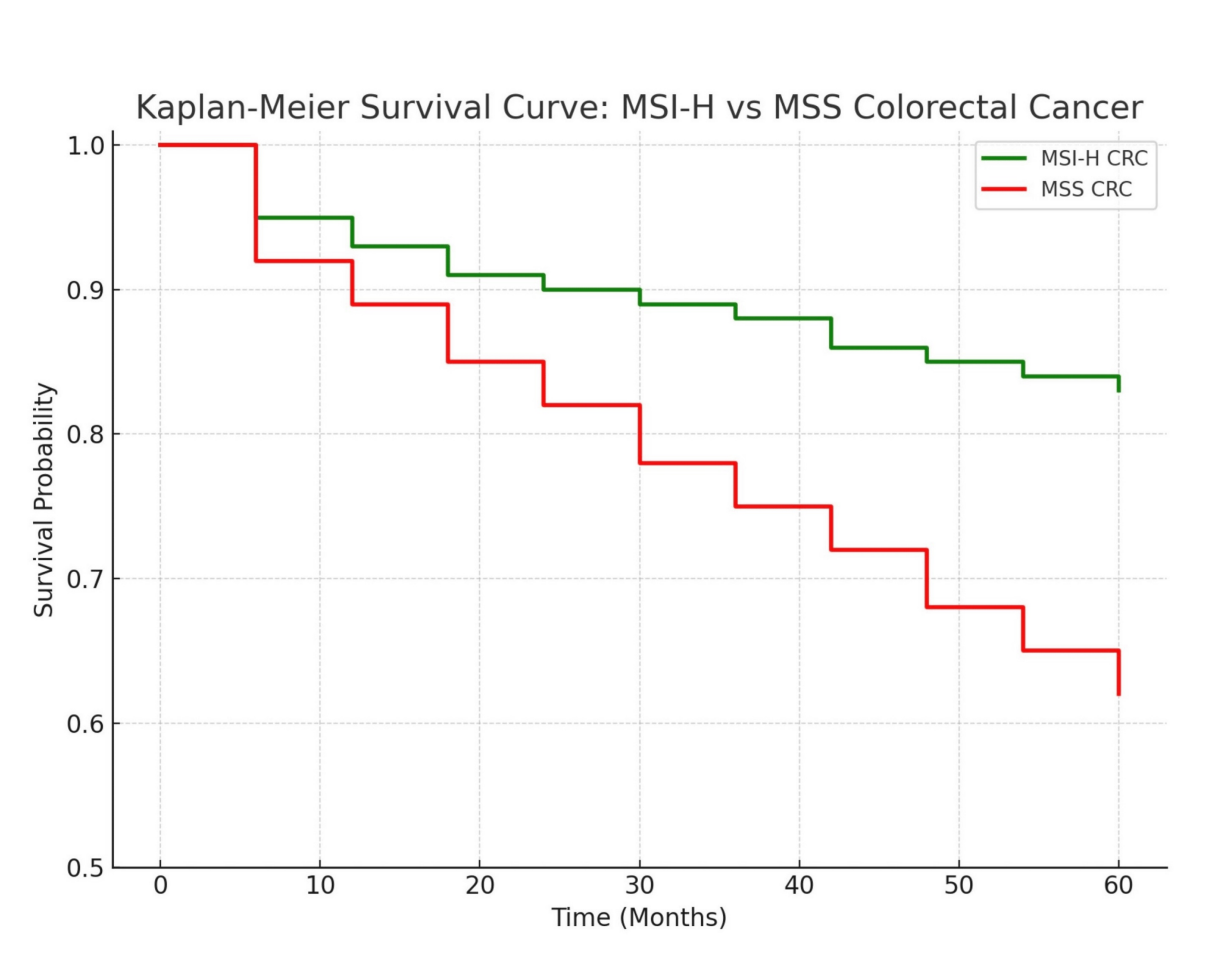
Kaplan-Meier survival curve comparing MSI-H and MSS colorectal cancer patients, demonstrating improved survival in the MSI-H group This survival curve was constructed using aggregate survival data from previously published studies evaluating MSI-H vs. MSS colorectal cancer outcomes [18-20]. MSI-H: microsatellite instability; MSS: microsatellite stable

The management of stage II CRC, particularly those with MSI-H status, is an area of active research and debate. Traditionally, adjuvant chemotherapy is not recommended for stage II patients without lymph node involvement. However, the role of adjuvant therapy in MSI-H tumors remains controversial, as these tumors are often resistant to conventional chemotherapy regimens such as fluoropyrimidines. Immunotherapy, on the other hand, may offer a promising treatment option, especially in cases where the tumor is resistant to traditional chemotherapeutic agents. Recent studies suggest that immune infiltration in MSI-H tumors contributes to their distinct immunologic profile, which may explain their responsiveness to immunotherapy [19-21]. Patients with MSI-H colorectal cancer often show better survival outcomes compared to MSS counterparts; this trend is clearly illustrated by Kaplan-Meier survival data reproduced from previous comprehensive studies analyzing these populations (Figure 5). Table 1 demonstrates the difference between the clinicopathological and molecular features of medullary carcinoma compared to conventional CRC.

**Table 1:** Clinicopathological and molecular features of medullary carcinoma vs. conventional CRC CRC: colorectal cancer

Feature	Medullary Carcinoma	Conventional CRC
Histology	Poorly differentiated, solid sheets, marked lymphocytic infiltration	Glandular differentiation, variable grade
Tumor Differentiation	Lack of glandular differentiation	Glandular differentiation
Microsatellite Instability (MSI)	MSI-H (deficient mismatch repair)	MSS or MSI-L (stable mismatch repair)
Lympho-Vascular Invasion	Present in 30-60% of cases	Present in 40-60% of cases
Perineural Invasion	Absent in most cases	Present in 40-60% of cases
Response to Immunotherapy	Better response to PD-1 inhibitors	Limited response to immune checkpoint inhibitors
Prognosis	Favorable with MSI-H status	Depends on the stage and molecular subtype
Typical Treatment	Surgical resection (mandatory for diagnosis), observation (postoperative surveillance in selected early-stage cases), or immunotherapy	Surgery and chemotherapy

Surveillance for colorectal cancer is crucial to detect early recurrence or metachronous lesions. Guidelines recommend serial carcinoembryonic antigen (CEA) testing, periodic imaging (CT scans), and colonoscopic evaluation to monitor for new lesions. Since this patient had no prior colonoscopic screening, early endoscopic evaluation was recommended. This case also highlights the need for individualized follow-up strategies based on the patient's age, comorbidities, and cancer characteristics. Close monitoring, including imaging and CEA levels, is essential to detect recurrence in the future [22-24]. Table 2 illustrates the follow-up plan for CRC.

**Table 2:** Follow-up plan CBC: Complete blood count, CMP: comprehensive metabolic panel, CEA: carcinoembryonic antigen, CT: computed tomography

Time point	Evaluation
6 months	CBC, CMP, CEA, CT Chest/Abdomen/Pelvis
12 months	Colonoscopy (if not done earlier), Repeat CT imaging
Ongoing	Annual CT imaging and clinical evaluation

Tumor markers like carcinoembryonic antigen (CEA) and carbohydrate antigen 19-9 (CA 19-9) are routinely utilized in the management of colorectal cancer for diagnosis, prognosis, and surveillance, but their use is limited in medullary carcinoma. Several studies have demonstrated that patients with MSI-H tumors, which also include medullary types, do not have persistently elevated CEA or CA 19-9 levels in even advanced disease. Thus, a value must be obtained with caution in these patients. Nonetheless, CEA continues to be part of standard surveillance, as in our patient. CA 19-9 is not standard but may be checked in some clinical situations, usually when pancreaticobiliary differentiation is considered [25].

## Conclusions

This case illustrates the importance of recognizing medullary carcinoma as a distinct entity within colorectal cancer. Given its MSI-H status, the absence of lymph node metastases, and the patient's age and comorbidities, a non-adjuvant approach with close surveillance was deemed appropriate. Long-term follow-up is crucial for early recurrence detection and management, and immunotherapy may play an increasingly significant role in the management of MSI-H colorectal cancer.
